# Voluntary versus mandatory food labels, Australia

**DOI:** 10.2471/BLT.24.291629

**Published:** 2024-08-27

**Authors:** Alexandra Jones, Damian Maganja, Maria Shahid, Bruce Neal, Simone Pettigrew

**Affiliations:** a The George Institute for Global Health, University of New South Wales, Level 18, International Towers 3, 300 Barangaroo Ave, Sydney NSW 2000, Australia.

## Abstract

**Objective:**

To compare uptake of the voluntary Health Star Rating front-of-pack nutrition labelling system with uptake of a mostly mandatory country-of-origin label in Australia over a similar period.

**Methods:**

We used data on numbers and proportions of products carrying health stars and country-of-origin labelling recorded annually between 2015 and 2023 through surveys of four large Australian food retailers. We determined the proportion of products with health stars and country-of-origin labels for each year by dividing the number of products carrying each label by the total number eligible to carry that label.

**Findings:**

The uptake of the voluntary Health Star Rating increased steadily between 2014 and 2018, reaching a maximum of 42% (8587/20 286) of products in 2021 before decreasing to 39% (8572/22 147) in 2023. Mandatory country-of-origin labelling uptake rose rapidly and was found on 93% (17 567/18 923) of products in 2023. In categories where country-of-origin labelling was voluntary, uptake by 2023 was 48% (3313/6925). In our 2023 sample of 22 147 products, 11 055 (50%) carried country-of-origin labelling only, 7466 (35%) carried both health star and country-of-origin labelling, 1106 (5%) carried health star labels only and 2520 (11%) carried neither label.

**Conclusion:**

The experience with country-of-origin labelling shows that widespread and rapid food labelling change can be achieved when required by law. The Australian government should mandate the Health Star Rating without delay. Australia’s experience supports other jurisdictions in implementing mandatory front-of-pack nutrition labelling as well as updates to global guidance to recognize mandatory labelling as best-practice in delivering benefits to consumers.

## Résumé

**Objectif:**

Comparer, au cours de la même période, l'adoption en Australie du système d'étiquetage nutritionnel facultatif Health Star Rating, à l'avant des emballages, avec celle de l'étiquetage mentionnant le pays d'origine, généralement obligatoire.

**Méthodes:**

Nous avons exploité les données relatives à la quantité et au pourcentage de produits arborant les étoiles du Health Star Rating et la mention du pays d'origine, récoltées sur une base annuelle entre 2015 et 2023 lors d'enquêtes menées auprès de quatre grands magasins d'alimentation australiens. Nous avons ensuite calculé le pourcentage de produits munis d'étoiles et d'une mention du pays d'origine pour chaque année en divisant le nombre de produits portant chaque étiquette par le nombre total de produits susceptibles d'en être pourvus.

**Résultats:**

L'adoption du système d'étiquetage nutritionnel facultatif Health Star Rating a augmenté de façon constante entre 2014 et 2018, parvenant à un maximum de 42% (8587/20 286) en 2021 avant de redescendre à 39% (8572/22 147) en 2023. Quant à l'étiquetage obligatoire concernant le pays d'origine, son adoption a rapidement grimpé pour atteindre 93% (17 567/18 923) des produits en 2023. Dans les catégories où la mention du pays d'origine n'était pas obligatoire, le pourcentage d'adoption en 2023 s'élevait à 48% (3313/6925). Dans notre échantillon de 2023, constitué de 22 147 produits, 11 055 (50%) n'indiquaient que le pays d'origine, 7466 (35%) présentaient les deux types d'étiquetage, 1106 (5%) n'affichaient que les étoiles du Health Star Rating et 2520 (11%) ne possédaient aucune des deux étiquettes.

**Conclusion:**

L'expérience menée sur la mention du pays d'origine montre qu'un changement d'étiquetage alimentaire rapide et largement répandu peut être obtenu par le biais d'une obligation légale. Le gouvernement australien doit donc imposer le système Health Star Rating au plus vite. Cette expérience encourage également d'autres juridictions à instaurer un étiquetage nutritionnel obligatoire à l'avant des emballages, ainsi qu'à mettre à jour les directives mondiales afin d'ajouter cette mesure aux bonnes pratiques bénéfiques pour les consommateurs.

## Resumen

**Objetivo:**

Comparar la adopción del sistema voluntario de etiquetado frontal nutricional Health Star Rating con la adopción de una etiqueta del país de origen mayoritariamente obligatoria en Australia durante un periodo similar.

**Métodos:**

Se utilizaron datos sobre el número y el porcentaje de productos que llevan etiquetas con estrellas de salud y el país de origen registrados cada año entre 2015 y 2023 a través de encuestas a cuatro de los principales minoristas de alimentos australianos. Se determinó el porcentaje de productos con etiquetas de estrellas de salud y del país de origen de cada año dividiendo el número de productos que llevaban cada etiqueta por el número total de productos elegibles para llevarla.

**Resultados:**

La adopción de la clasificación voluntaria Health Star Rating aumentó de forma constante entre 2014 y 2018, alcanzando un máximo del 42% (8587/20 286) de los productos en 2021 antes de disminuir al 39% (8572/22 147) en 2023. El etiquetado obligatorio del país de origen aumentó rápidamente y se encontró en el 93% (17 567/18 923) de los productos en 2023. En las categorías en las que el etiquetado del país de origen era voluntario, la adopción en 2023 fue del 48% (3313/6925). En la muestra de 2023, compuesta por 22 147 productos, 11 055 (50%) solo llevaban el etiquetado del país de origen, 7466 (35%) llevaban tanto el etiquetado de las estrellas de salud como el del país de origen, 1106 (5%) solo llevaban el etiquetado de las estrellas de salud y 2520 (11%) no llevaban ninguna de estas etiquetas.

**Conclusión:**

La experiencia con el etiquetado del país de origen demuestra que se puede conseguir un cambio rápido y generalizado en el etiquetado de los alimentos cuando así lo exige la ley. El gobierno australiano debería exigir la clasificación Health Star Rating sin demora. La experiencia de Australia apoya a otras jurisdicciones en la implementación del etiquetado frontal nutricional obligatorio, así como en la actualización de las directrices mundiales para reconocer esta medida como una de las mejores prácticas en beneficio de los consumidores.

## ملخص

**الغرض:**

مقارنة بين استخدام نظام ملصقات المعلومات الغذائية الطوعي على واجهة العبوات، والحاصل على تصنيف نجوم الصحة (Health Star Rating)، في مقابل استخدام ملصق بلد المنشأ الإلزامي غالبًا في أستراليا خلال فترة مشابهة.

**الطريقة:**

قمنا باستخدام بيانات حول أعداد ونسب المنتجات التي تحمل تصنيف نجوم الصحة، وملصقات بلد المنشأ المسجلة سنويًا بين عامي 2015 و2023 من خلال المسوح التي تمت على أربعة من كبار تجار التجزئة للأغذية في أستراليا. حددنا نسبة المنتجات ذات تصنيف نجوم الصحة وملصقات بلد المنشأ لكل عام عن طريق تقسيم عدد المنتجات التي تحمل كل ملصق على العدد الإجمالي المؤهل لحمل هذا الملصق.

**النتائج:**

زاد استخدام تصنيف النجوم الصحي الطوعي بشكل مطرد بين عامي 2014 و2018، وبلغ ذروته عند %42 (8587/20286) من المنتجات في عام 2021 قبل أن ينخفض إلى %39 (8572/22147) في عام 2023. ارتفع استخدام ملصق بلد المنشأ الإلزامي بسرعة، وتم العثور عليه على %93 (17567/18923) من المنتجات في عام 2023. وفي الفئات التي كان فيها ملصق بلد المنشأ طوعيًا، بلغ الاستخدام بحلول عام 2023 نسبة %48 (3313/6925). في عينة عام 2023 التي شملت 22147 منتجًا، حمل 11055 منتجًا (%50) ملصق بلد المنشأ فقط، وحمل 7466 منتجًا (%35) كل من ملصق نجمة الصحة وملصق بلد المنشأ، وحمل 1106 منتجًا (%5) ملصق نجمة الصحة فقط، ولم يحمل 2520 منتجًا (%11) أي ملصق.

**الاستنتاج:**

توضح التجربة مع ملصق بلد المنشأ أنه يمكن تحقيق تغيير واسع النطاق وسريع في ملصقات الغذاء عندما يتطلب القانون ذلك. يجب على الحكومة الأسترالية أن تفرض تصنيف نجمة الصحة دون تأخير. تدعم تجربة أستراليا الولايات القضائية الأخرى في تنفيذ ملصقات التغذية الإلزامية على واجهة العبوات، فضلاً عن تحديث الإرشادات العالمية للاعتراف بهذا الإجراء باعتباره أفضل ممارسة لمصلحة المستهلكين.

## 摘要

**目的:**

比较澳大利亚在同一时期采用包装正面自愿性标识“健康星级评价”营养标签体系和采用大部分为强制性的原产国标签的情况。

**方法:**

我们使用 2015 年至 2023 年期间每年记录的标识了健康星级和原产国标签的产品数量和比例数据,对澳大利亚四家大型食品零售商进行了调查。通过将标识了健康星级和原产国标签的产品数量除以符合表标识该标签条件的产品总数,我们确定了每年标识健康星级和原产国标签的产品比例。

**结果:**

2014 至 2018 年期间,标识“健康星级评价”标签的自愿采用率稳步上升,在 2021 年达到最高值 42% (8,587/20,286),然后在 2023 年降至 39% (8,572/22,147)。强制性原产国标签的采用率迅速上升,2023 年有 93% (17,567/18,923) 的产品上都发现了这一点。到 2023 年,对自愿性原产国标签的采用率为 48% (3,313/6,925)。在我们纳入 2023 年样本的 22,147 件产品中,有 11,055 件 (50%) 仅标识了原产地标签,7,466 件 (35%) 同时标识了健康星级和原产国标签,1,106 件 (5%) 仅标识了健康星级标签,而有 2,520 件 (11%) 未标识任何标签。

**结论:**

对原产国标签的采用情况表明,只要法律要求,就可以实现广泛而快速的食品标签变革。澳大利亚政府应立即强制要求采用“健康星级评价”标签。澳大利亚的经验有助于其他司法管辖区强制强制要求包装正面标识营养标签并更新全球指南,从而将这一措施视为维护消费者利益的最佳实践。

## Резюме

**Цель:**

Сравнить внедрение системы добровольной маркировки пищевых продуктов с рейтингом Health Star Rating на упаковке с использованием обязательной маркировки страны происхождения в Австралии за аналогичный период.

**Методы:**

Были использованы данные о количестве и доле продуктов со звездами здоровья и маркировкой страны происхождения, которые ежегодно регистрировались в период с 2015 по 2023 год в ходе опросов четырех крупных австралийских компаний розничной торговли продуктами питания. Для определения доли продуктов с маркировкой «звезды здоровья» и «страна происхождения» за каждый год количество продуктов с каждой маркировкой делится на общее количество продуктов, претендующих на эту маркировку.

**Результаты:**

В период с 2014 по 2018 год доля продуктов, добровольно использующих рейтинг Health Star Rating, неуклонно росла, достигнув максимума в 42% (8587/20 286) в 2021 году, а затем снизившись до 39% (8572/22 147) в 2023 году. Использование обязательной маркировки страны происхождения быстро росло, и в 2023 году она была указана на 93% (17 567/18 923) продуктов. В категориях, где маркировка страны происхождения являлась добровольной, ее использование к 2023 году составило 48% (3313/6925). В выборке 2023 года из 22 147 продуктов на 11 055 (50%) была указана только страна происхождения, на 7466 (35%) – и звезда здоровья, и страна происхождения, на 1106 (5%) – только звезда здоровья, а на 2520 (11%) не было указано ни одной из этих маркировок.

**Вывод:**

Опыт маркировки страны происхождения свидетельствует о том, что повсеместное и быстрое изменение маркировки продуктов питания может осуществляться, если этого требует закон. Правительству Австралии следует незамедлительно сделать обязательным рейтинг «звезд здоровья» (Health Star Rating). Опыт Австралии способствует внедрению обязательной маркировки продуктов питания на лицевой стороне упаковки в других юрисдикциях, а также обновлению глобального руководства, чтобы признать эту меру в качестве передового опыта, направленного на благо потребителей.

## Introduction

Unhealthy diets are a leading cause of death and disability globally.^1^ Current food environments are characterized by unprecedented availability, accessibility and affordability of prepackaged and often ultraprocessed foods and beverages (hereafter called foods). These foods are a key driver of obesity and diet-related noncommunicable diseases including high blood pressure, cardiovascular disease, type 2 diabetes and some cancers.^2^^,^^3^ Governments can help reduce the burden of diet-related noncommunicable diseases by implementing policies to create healthier food environments, including actions to improve food labelling.^4^^,^^5^

Food labels are a contested space. For manufacturers, labels represent marketing space. For governments, public health stakeholders and consumer groups, food labelling is a potential public policy tool. Simple, graphical, front-of-pack nutrition labels provide at-a-glance information on nutritional quality on the main display panel of foods to complement detailed nutrient declarations on the back of the pack.^6^ Recent systematic reviews suggest front-of-pack nutrition labelling can improve consumer understanding of the nutritional quality and content of foods, and improve the healthiness of food choices and purchases.^7^ Interpretive front-of-pack nutrition labelling (using meaningful symbols, words and colours) have a greater effect on these outcomes than non-interpretive systems (using primarily numerical information).^8^ Front-of-pack nutrition labelling may also provide incentives for the food industry to reformulate their products to improve their nutritional quality.^6^^,^^9^

More than 32 governments have implemented some form of front-of-pack nutrition labelling.^10^^,^^11^ Policy developments include a shift away from so-called softer positive signposts that highlight healthier options, such as Sweden’s Keyhole,^12^ towards innovation in two directions. Led by the World Health Organization (WHO) Region of the Americas, 10 countries have followed Chile’s lead in adopting so-called stop-sign warnings^13^ that signal products high in risk nutrients and discourage consumption. Elsewhere, Australasia and several European countries have adopted systems that rate foods overall on a spectrum with potential to signal both unhealthy and healthier choices.^14^^,^^15^ Evidence is inconsistent on which of these interpretive front-of-pack nutrition labelling systems performs best.^7^

Global guidance encourages government action but allows policy-makers to determine the content of national regulation.^6^^,^^16^^–^^18^ WHO recommends front-of-pack nutrition labelling as a best-buy policy to reduce unhealthy diets in its interventions to address noncommunicable diseases.^5^ More specifically, the WHO *Guiding principles and framework manual for front-of-pack labelling for promoting healthy diets* sets out best practices for countries looking to develop, implement, monitor and evaluate effective front-of-pack nutrition labelling.^6^ The international food standards agency, the Codex Alimentarius Commission (Codex), also adopted new guidelines for front-of-pack nutrition labelling in 2021 to provide direction on developing front-of-pack nutrition labelling.^17^ Both documents allow governments to select which label format will be most effective for their populations. They also recognize that a key policy decision for governments is whether front-of-pack nutrition labelling should be implemented on a voluntary or mandatory basis, but neither makes explicit recommendation on which will work best.^6^^,^^17^

Making any new food label mandatory depends somewhat on what is legally possible in a jurisdiction. For example, in the European Union (EU), regulation restricts EU members from implementing mandatory labels that create barriers to free movement of food between EU countries.^19^ Although obligations under international trade law are sometimes raised as a challenge to mandatory labelling, decisions of dispute resolution bodies of the World Trade Organization confirm that countries can implement mandatory labels where necessary to protect public health, consumers and/or the environment.^20^^–^^22^ At least 10 countries have already implemented some form of mandatory front-of-pack nutrition labelling.^10^

In jurisdictions where mandatory labels are legally feasible, determining whether they will be adopted still requires policy-makers to balance potential costs and benefits to different stakeholders. From the perspective of achieving health, social or environmental outcomes, mandatory labels provide consumers with wider access to information and the opportunity to make comparisons between foods in a way that encourages improved purchasing and consumption. Mandatory labelling may also provide stronger incentives for industry to reformulate their products.^23^ By contrast, food industry stakeholders may oppose mandatory labels where these jeopardize profits, for example by increasing scrutiny on unhealthy products.^24^ Industry stakeholders may also cite the costs of redesigning and reprinting packaging as an argument against labelling reform, particularly if updates are required quickly.^22^^,^^25^ In Australia, this balancing can be seen in the outcomes of two recent food-labelling reforms: the government’s decisions to adopt the Health Star Rating front-of-pack nutrition label on a voluntary basis, and to require a new country-of-origin label on a mandatory basis for most foods.

In 2014, the Australian and New Zealand governments adopted the Health Star Rating system as a form of front-of-pack nutrition labelling.^15^ This system uses an algorithm to summarize the nutritional quality of foods and assign an overall rating from 0.5 stars (least healthy) to 5.0 stars (most healthy) in 10 half-star increments (online repository).^26^ By rating food on a spectrum, this system has the potential to both encourage and/or discourage consumption.^10^

At adoption, the food ministers agreed the Health Star Rating could remain voluntary subject to “consistent and widespread uptake.”^27^ By 2019, the Health Star Rating was on 41% (7118/17 477) of eligible products, mostly those that had good star ratings.^28^ While public health and consumer groups have advocated that the government make the Health Star Rating mandatory,^29^ manufacturer groups continue to strongly oppose this move.^30^ In July 2020, food ministers finalized the outcomes of a formal 5-year review of the system and concluded it could remain voluntary for 5 more years.^31^ The ministers also held that if this system continued to perform well but was not displayed on > 70% of target products by 2025, they would consider converting to a mandatory system.^32^

In contrast to its voluntary approach to front-of-pack nutrition labelling, in July 2016, the Australian government implemented new mandatory country-of-origin labelling. Regulations require most food suitable for retail sale in Australia to carry a country-of-origin label, namely a text statement and/or graphic label known as a standard mark.^33^ How country-of-origin labels are displayed depends on whether the food was grown, produced, made or packed in Australia or another country (online repository).^26^ The purpose of this label was to provide clear, consistent and informative country-of-origin information for food so that consumers could make more informed choices in line with personal preferences.^34^ In supporting documents, the government recognized that consumer preferences varied and that country-of-origin labelling may serve as a proxy for information on food quality, safety, traceability and healthiness. Such labelling may also signal that a product supports national jobs, manufacturing and the local economy.^34^

For most products, deemed so-called priority products under the regulation, a country-of-origin label is mandatory, with compliance required by 1 July 2018. For limited categories of so-called non-priority foods where consumers are purportedly less interested in country-of-origin information,^35^ this label remains voluntary for manufacturers to display. Businesses that do not comply with the country-of-origin labelling can be held in breach of Australian law and risk considerable financial penalties.^36^ In July 2021, a government evaluation found that country-of-origin label reforms were well implemented overall.^35^

The implementation of the Health Star Rating and country-of-origin label policies over similar time periods provides a natural experiment for assessing labelling compliance resulting from differing regulatory approaches. To that end, we compared the food industry’s use of mandatory country-of-origin labelling versus voluntary Health Star Rating in Australia between 2015 and 2023.

## Methods

We examined uptake of the Health Star Rating and country-of-origin label using repeated cross-sectional surveys of the Australian packaged food supply. The George Institute’s FoodSwitch programme captures images of packaged foods via a bespoke mobile application, which allows extraction and collation of labelling and composition data.^37^ FoodSwitch monitoring data sets are generated annually based on systematic data collection from four large Australian retailers (Aldi, Coles, Independent Grocers of Australia and Woolworths) in metropolitan Sydney. Trained data collectors visit stores between March and April annually to photograph the labels of all packaged foods and beverages available in-store, and trained data-entry personnel extract key information from the images captured, including the presence or absence of the health stars and country-of-origin labelling. Products captured in any year are estimated to account for 90% of packaged foods in Australia based on sales.^38^

We used FoodSwitch monitoring data sets from 2015 to 2023 to compare annual uptake of the health stars and country-of-origin labelling, noting that no collection was undertaken in 2020 due to the coronavirus disease 2019 (COVID-19) pandemic.

Data collectors identified products using their barcodes. Where a product appeared in more than one size (e.g. 375 mL can and 600 mL bottle of the same drink), each size was counted as an individual product. This approach captures the number of unique product packages updated by manufacturers to incorporate new labelling requirements.

### Eligibility for food labelling

FoodSwitch classifies foods and beverages into a hierarchical category tree.^37^ Products are categorized into major categories (e.g. bread and bakery products), minor categories (e.g. biscuits and bread) and further subcategories.

For both the Health Star Rating system and country-of-origin label, eligibility is mainly determined by product category. All products are eligible for the health stars unless they fall into excluded categories listed in the Health Star Rating guide, which are: alcohol; formulated supplementary sports foods; infant foods and formulas; foods for special medical purposes; and vitamins and supplements.^39^ Thus, 15 major food categories were available for analysis. Within these categories, we also excluded minor categories such as baking sodas and powders, chewing gum, herbs and spices, plain teas and coffees, yeasts and gelatines. We excluded these foods as they do not contribute significantly to nutrient intake and are not required to carry a nutrient information panel. While unpackaged foods (e.g. fresh fruit and vegetables) and some other products are not required to display a nutrient information panel, they are permitted to display the Health Star Rating (e.g. plain bottled water, unprocessed raw meat, poultry and fish). However, these foods are excluded from the government’s uptake targets for health-star labelling; as such we also excluded these products from our analyses.

We considered all products eligible for country-of-origin labelling except vitamins and supplements, which are explicitly excluded in the regulations.^33^ We separated products eligible for country-of-origin labelling into priority and non-priority products as specified by the regulations. All products were priority products except those in the following categories: alcohol; beverage mixes; biscuits; coffee and tea; confectionery; cordials; crisps and snacks; dessert toppings; electrolyte drinks; energy drinks; herbs and spices; ice cream and edible ices; ice cream cones; soft drinks; sports powders and gels; and waters (plain and flavoured).^33^ Country-of-origin labelling is mandatory for priority products, whereas it is voluntary for non-priority products.

### Uptake of food labelling

The presence or absence of the Health Star Rating has been routinely determined since 2015 by examining images for one of five variants of the labelling (online repository).^26^ FoodSwitch continues to record the use of these five variants (four variants use the star graphic and one uses a so-called energy icon only). However, after November 2020, the energy icon only became invalid. We assumed that use of health stars was 0% in 2014, the year of its introduction. We flagged a product at data entry from 2017 as carrying an updated country-of-origin label if it displayed any of the permitted variants within the regulation (online repository).^26^ We assumed use of the new country-of-origin labelling was 0% in 2016, the year of its introduction.

### Statistical analyses

For the primary analysis, we determined uptake of health stars on eligible products and uptake of country-of-origin labelling on priority and non-priority products separately for each year from 2015 to 2023 by dividing the number of products carrying each label by the total number eligible to carry that label. For 2021 to 2023, uptake of health stars included both products displaying the star graphic and products continuing to display the energy icon only, as the government allowed a transition period for this change. Additional data on products displaying the energy icon-only variant are provided in the online repository.^26^

To understand how manufacturers chose to apply the two labels to their products, we also examined the absolute number of products carrying only health stars, only the country-of-origin label, or both labels each year. Only products eligible to carry both labels were considered in this secondary analysis. We undertook all analyses in Stata/BE version 18.0 (StataCorp. LP, College Station, United States of America).

## Results

In 2023, 22 147 products were eligible to show health stars and 25 848 products were eligible for a country-of-origin label. Of the latter products, 18 923 were priority products for country-of-origin labelling and 6925 were non-priority products.

**Fig. 1:**
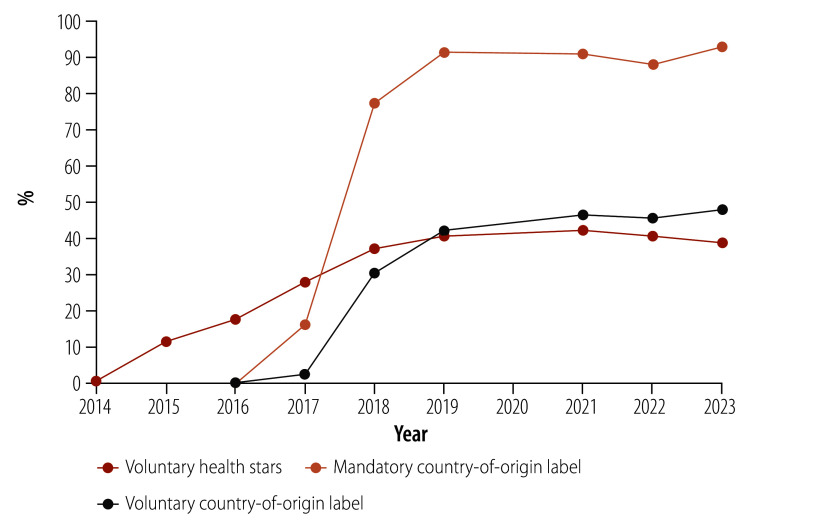
Percentage of eligible food products displaying health stars, the mandatory country-of-origin label and voluntary country-of-origin label, Australia, 2014–2023

Uptake of each label over time as a proportion of eligible products is shown in Fig. 1 and Table 1. Voluntary uptake of health stars increased steadily between 2014 and 2018, before plateauing at a maximum of 42% (8587/20 286) in 2021 and decreasing to 39% (8572/22 147) in 2023. By 2023, country-of-origin labels were on 93% (17 567/18 923) of priority products where they are mandatory, and 48% (3313/6925) of non-priority products where they are voluntary.

**Table 1:** Eligible food products displaying health stars, the mandatory country-of-origin label and voluntary country-of-origin label, Australia, 2015–2023

Year	No./total (%)		
	Voluntary health stars	Mandatory country-of-origin label	Voluntary country-of-origin label
2015	1843/16122 (11)	NA/NA (NA)	NA/NA (NA)
2016	2840/16192 (18)	NA/NA (NA)	NA/NA (NA)
2017	4360/15782 (28)	2179/13764 (16)	9572/208 (2)
2018	6942/18659 (37)	12337/15974 (77)	5984/1826 (31)
2019	7120/17548 (41)	13846/15134 (91)	5599/2352 (42)
2021	8587/20286 (42)	15407/16938 (91)	6277/2927 (47)
2022	8518/20946 (41)	16706/18951 (88)	6603/3012 (46)
2023	8572/22147 (39)	17567/18923 (93)	6925/3313 (48)

NA: not applicable.

Note: the Health Star Rating system was introduced in 2014 and country-of-origin labelling was introduced in 2016. We assumed 0% uptake in the year of introduction.

Removal of products carrying the energy icon-only label reduced health star uptake to 37% across 2021 (7543/20 286), 2022 (7801/20 946) and 2023 (8209/22 147; online repository),^26^ although progressively fewer (5%; 1044/20 286; 3%; 717/20 946; and 2%; 363/22 147) products displayed the energy icon only each year, suggesting this label was being phased out. Without the energy icon-only label, use of health stars only rose by 6% (from 31%; 5777/18 659 to 37%; 8209/22 147) between 2018 and 2023.

**Fig. 2:**
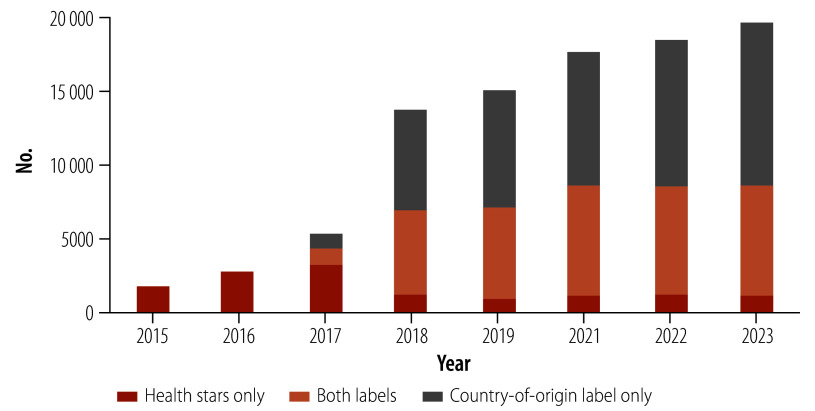
Number of food products displaying health stars only, the country-of-origin label only and both labels, Australia, 2015–2023

As all products eligible for the Health Star Rating are also eligible to display a country-of-origin label, a total of 22 147 products were eligible for both labels. Fig. 2 illustrates the pattern of manufacturers’ changes to packaging eligible to carry both labels. In 2023, 34% (7466/22 147) of products carried both health stars and country-of-origin labelling, while for 50% (11 055/22 147) of products, manufacturers had changed the label to display country-of-origin while electing not to add health stars. The corresponding figure for products carrying health stars only was 5% (1106/22 147), and 11% (2520/22 147) of eligible products carried neither label.

## Discussion

High and rapid compliance with mandatory country-of-origin labelling requirements demonstrates the feasibility of food manufacturers updating product labels when required by law. These results are in contrast with the limited uptake of voluntary labelling for both the Health Star Rating and country-of-origin for non-priority products.

Our findings indicate that many manufacturers are electing to withhold front-of-pack nutrition labelling, even when they have updated product packaging to display a new country-of-origin label. This finding suggests that the cost of redesigning and updating packaging is not the main factor influencing company behaviour, even though the industry frequently raises this issue as a justification for lenience from governments in applying new labels.^25^^,^^40^^,^^41^

After nearly 10 years of voluntary implementation, plateauing and slightly declining uptake of health stars is disappointing. Our finding of poor ongoing uptake overall builds on previous research demonstrating that uptake of this system is skewed heavily towards higher-scoring products while remaining absent from most low-scoring foods.^42^ Both results suggest most manufacturers are only likely to display health stars voluntarily when they confer marketing benefit. Unless immediate action is taken to increase uptake on unhealthy foods, the Australian Government’s target of > 70% uptake of Health Star Rating on food products by 2025 will not be achieved.

In any future consideration of whether the Health Star Rating system should be made mandatory, Australian food ministers will need to conduct further analysis of costs and benefits.^43^ Our findings contribute to this balancing exercise by providing data on the failure of voluntary labelling to build the case for increasing government intervention.^44^^,^^45^ Our data on the existing proportion of products carrying health stars can help inform the scope and cost of the remaining changes required by industry. Finally, our findings on the rapid uptake of country-of-origin labelling demonstrate the feasibility of a 2–3-year transition period for labelling change.

Beyond Australia, our findings provide lessons for policy-makers interested in maximizing the impact on public health of front-of-pack nutrition labelling. Our results provide strong justification for mandating the use of interpretive front-of-pack nutrition labelling that are currently voluntary. For countries developing front-of-pack nutrition labelling systems, our work suggests that adopting mandatory requirements from the outset will deliver benefits to consumers more rapidly.

Our findings can inform future decision-making by governments on whether and how to engage with the food industry on government-led measures to promote healthier diets. Unlike Article 5.3 of the WHO Framework Convention on Tobacco Control, there is no requirement for governments to exclude food industry stakeholders from policy development,^46^ although WHO has developed several tools which recognize the need to safeguard nutrition policy-making from conflicts of interest.^47^^,^^48^ Australia’s experience with the Health Star Rating system suggests little value in maintaining voluntary front-of-pack nutrition labelling in collaboration with the food industry. While adopting mandatory, government-led legislation may require strong political will, maintaining voluntary initiatives also requires human and financial resources from the government, with the risk of fewer public health and consumer benefits.

As real-world evidence grows, global normative guidance such as forthcoming WHO guidelines on nutrition labelling^49^ needs to recognize that front-of-pack nutrition labelling should be applied universally to deliver maximum benefits. Broad statements that front-of-pack nutrition labelling can be voluntary or mandatory are not sufficient to guide best practice in health policy. Recognition that front-of-pack nutrition labelling can be compulsory provides countries implementing mandatory labels with a useful defence in the event of legal challenge.^22^ Legal feasibility should not be overstated as a barrier to mandatory labels, particularly given updated Codex guidance that recognizes mandatory front-of-pack nutrition labelling is aligned with international standards.^17^ In common markets such as the EU or the East African Community that have rules restricting member countries from adopting country-specific mandatory labels, harmonized mandatory front-of-pack nutrition labelling could offer a legally feasible solution that balances the interests of health and trade. For instance, our findings point to the benefits that could be realized in the EU if the European Commission finalizes its overdue proposal for harmonized mandatory front-of-pack nutrition labelling^50^ by, for example, adopting legislation to mandate the Nutri-Score system which is currently voluntary in Belgium, France and Spain.^14^

A strength of our study is its use of a high-quality longitudinal data set covering a large sample of products to illustrate differences in implementation of two policies in the same market, by the same manufacturers and over the same period. As a natural experiment, we acknowledge that other variables may affect the use of labels, e.g. different placement requirements on packs. However, our findings, including similar results for Health Star Rating system and non-priority (voluntary) country-of-origin labelling, clearly suggest that our approach provided a robust test of the effect of implementing voluntary versus mandatory labelling. However, our unique policy context may limit the transferability of the methods to other settings. While some errors may have arisen in our assignment of individual products as eligible for labelling under each system, the differences in uptake of each system are large and hence our conclusions about the importance of mandatory labelling are not likely to be affected. Likewise, while the data set does not include every food product in Australia eligible for each labelling system, it does cover the great majority of the most widely purchased products.

Finally, no single policy action will be sufficient to tackle the global burden of unhealthy diets. Consistent with existing normative guidance, policy-makers should frame labelling reforms as part of a suite of comprehensive policies; ensure labelling reform is aligned with dietary guidelines and other nutrition policies; and provide complementary consumer education that contextualizes the usefulness of labelling reform within healthy dietary patterns and foods.^6^^,^^17^ To extend the usefulness of labelling reforms, authorities may also consider integrating labelling reform with measures on, for example, restrictions on marketing of unhealthy foods, school food and other public procurement policies to stimulate greater effects across the food system.^10^
